# Low prevalence of fibrosis in thalassemia major assessed by late gadolinium enhancement cardiovascular magnetic resonance

**DOI:** 10.1186/1532-429X-13-8

**Published:** 2011-01-17

**Authors:** Paul Kirk, John Paul Carpenter, Mark A Tanner, Dudley J Pennell

**Affiliations:** 1 Royal Brompton Hospital and Imperial College, London, UK

## Abstract

**Background:**

Heart failure remains a major cause of mortality in thalassaemia major. The possible role of cardiac fibrosis in thalassemia major in the genesis of heart failure is not clear. It is also unclear whether cardiac fibrosis might arise as a result of heart failure.

**Methods:**

We studied 45 patients with thalassaemia major who had a wide range of current cardiac iron loading and included patients with prior and current heart failure. Myocardial iron was measured using T2* cardiovascular magnetic resonance (CMR), and following this, late gadolinium enhancement (LGE) was used to determine the presence of macroscopic myocardial fibrosis.

**Results:**

The median myocardial T2* in all patients was 22.6 ms (range 5.3-58.8 ms). Fibrosis was detected in only one patient, whose myocardial T2* was 20.1 ms and left ventricular ejection fraction 57%. No fibrosis was identified in 5 patients with a history of heart failure with full recovery, in 3 patients with current left ventricular dysfunction undergoing treatment, or in 18 patients with myocardial iron loading with cardiacT2* < 20 ms at the time of scan.

**Conclusion:**

This study shows that macroscopic myocardial fibrosis is uncommon in thalassemia major across a broad spectrum of myocardial iron loading. Importantly, there was no macroscopic fibrosis in patients with current or prior heart failure, or in patients with myocardial iron loading without heart failure. Therefore if myocardial fibrosis indeed contributes to myocardial dysfunction in thalassemia, our data combined with the knowledge that the myocardial dysfunction of iron overload can be reversed, indicates that any such fibrosis would need to be both microscopic and reversible.

## Introduction

Thalassaemia is the commonest single gene disorder worldwide, with approximately 94 million heterozygotes for beta thalassaemia and 60,000 homozygotes born each year [1]. Life-long blood transfusions are required for survival in thalassemia major, but with each unit of blood carrying 200-250 mg of iron, the transfusions result in tissue iron loading and multiple organ complications. Myocardial siderosis is the major cause of mortality and is dominantly manifested as heart failure [2], which typically has a disguised onset that may deteriorate catastrophically as a result of a vicious cycle of increasing intracellular iron levels. This toxic cardiomyopathy can be reversible if chelation is commenced early [3], although it may take years to reduce cardiac storage iron levels to normal. Although heart failure is clearly related to intracardiac iron, it is less clear whether there is an additional contribution from myocardial fibrosis. Myocardial fibrosis could reduce both systolic function and ventricular compliance which could aggravate iron mediated cardiac dysfunction. Another possibility is that cardiac fibrosis might occur as a result of periods of cardiac failure, and therefore its occurrence might indicate previous cardiac damage with recovery. Historical papers have suggested that cardiac fibrosis is a major pathological component of heart failure and death in cardiac siderosis [4-6], but this data is up to 50 years old and may be misleading in the current era of iron chelation treatment. There is rather little contemporary data examining the histological incidence of fibrosis in cardiac iron overload [7-9], and its relation to ventricular function and iron levels in thalassaemia patients [10].

Cardiac fibrosis can be studied using late gadolinium enhancement (LGE) cardiovascular magnetic resonance (CMR). This technique uses an injection of a gadolinium chelate which is an MR contrast agent that concentrates in areas of expanded extracellular space, such as myocardial fibrosis, from 10 minutes after injection. LGE has been widely used to examine the role of replacement macroscopic fibrosis in a range of cardiomyopathies [11-14], and the occurrence of LGE is associated with the development of cardiac events, such as heart failure and arrhythmias [15-17]. CMR has also been used widely to measure storage cardiac iron with the T2* technique [18], which is non-invasive, reproducible [19,20], calibrated to cardiac iron levels [21-23], and a valuable technique to assess the efficacy of cardiac iron chelators [3,24-26]. We therefore used LGE and cardiac T2* to investigate the incidence of fibrosis and its correlation with myocardial iron and myocardial function in thalassaemia major patients.

## Methods

### Patients

A total of 45 patients with beta-thalassaemia major were included in this study. Patients were referred for clinical evaluation according to local practice at external referring centers. There were 19 male and 26 female patients with a mean (±SD) age of 27.1 ± 9.6 years. This research had full ethical approval through Trent NHS Ethics Committee, and all patients gave written consent.

### Cardiovascular Magnetic Resonance

Patients were scanned at 1.5 T (Sonata, Siemens Medical Solutions, Erlangen, Germany) using previously reported techniques [18]. In brief, T2* CMR was performed using a cardiac gated, single breath-hold, 8-echo sequence (2.6 - 16.7 ms, with 2.02 ms increments) of a single mid-ventricular short axis slice. Long axis cines and a contiguous stack of short axis cines were also acquired to assess left ventricular dimensions and function [27]. For T2* acquisition a large region of interventricular septum in a mid-ventricular slice was used. Following T2* acquisition, intravenous gadolinium-DTPA (0.2 mmol/kg) was administered and LGE images were obtained in multiple planes after 10 minutes [28]. Multiple acquisitions using an inversion recovery-prepped gradient echo sequence were obtained with incrementally increased inversion times (TI) until optimal contrast between normal and abnormal myocardium was obtained with TI = 250 ms. Data analysis was performed using CMRtools and its plug-in Thalassaemia Tools (Cardiovascular Imaging Solutions, London UK). All scans were reported in consensus by two independent experienced CMR readers.

### Statistics

T2* values do not conform to a Gaussian distribution and are therefore presented as medians (Q1, Q3) unless otherwise stated. Comparison of T2* readings were made using the Mann-Whitney U test and comparisons of left ventricular indices were performed using Student's *t *test. All normal variables are presented as mean ± SD and ranges or n (%) as appropriate. Statistical significance was set at P < 0.05.

## Results

### Overall patient cohort details

The baseline characteristics of the whole study group are shown in table 1. There were 42 subjects with normal left ventricular (LV) ejection fraction (EF > 56%) and 3 patients with current LV impairment with LVEF < 56% who were receiving cardiac treatment for heart failure. Five patients (11%) had a previous history of heart failure and 4 patients (9%) had history of documented arrhythmias, all of whom currently has normal LV function and no heart failure. The median (Q1, Q3) of myocardial T2* in all patients was 22.6 ms (range 5.3-58.8 ms). Of these patients, 27 had no significant iron loading (T2* > 20 ms), 15 patients had mild/moderate iron loading (T2* 10-20 ms) and 3 patients had severe iron loading (T2* < 10). At the time of the CMR scan, 1 patient was receiving no chelation (post bone marrow transplant), 21 patients were receiving deferoxamine, 3 patients deferiprone, 11 patients deferoxamine combined with deferiprone and 9 patients deferasirox only.

**Table 1:** Demographics (mean ± SD unless otherwise stated).

Total number of patients	45
**Age (years)**	27.1 ± 9.6

**Sex**	
Male	19
Female	26

**Race/ethnicity**	
White	19
South Asian	13
Arabic	8
African	5

**Units of blood transfused (units/year)**	32.2 ± 17.3

**Past history cardiac disease**	
Heart Failure	5 (11%)
Time between heart failure and scan (years)	3.7 ± 7.6
Arrhythmia	4 (9%)
Time between arrhythmia and scan (years)	4.0 ± 6.7

**CMR measures:**	
Cardiac T2* (ms) median (Q1, Q3)	22.6 (13.4, 34.5)
Liver T2* (ms) median (Q1, Q3)	4.4 (2.3, 8.5)
LV end diastolic volume (mL)	134 ± 41.4
LV end systolic volume (mL)	46.4 ± 20.2
LV ejection fraction (%)	65.8 ± 8.5

**Chelation**	
Deferoxamine only	21 (47%)
Deferiprone only	3 (7%)
Deferasirox only	9 (20%)
Deferoxamine & deferiprone	11 (24%)
No Chelation	1 (2%)

### No myocardial iron loading group (T2* > 20 ms)

**Figure 1:**
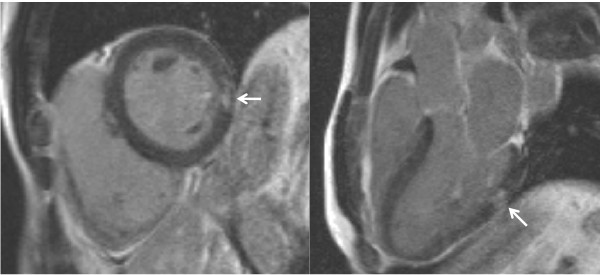
**Late gadolinium enhancement in the thalassaemia patient. **The arrows show a small area of fibrosis in the mid inferolateral wall of the left ventricle.

In these 27 patients, the myocardial T2* was 32.0 ms (26.5, 39.3), liver T2* was 6.8 ms (4.4, 21.0) and the LV EF was 68.3 ± 8.3%. No patient had a prior history of heart failure. There was fibrosis in one patient, (T2* 20.1 ms, and LVEF 57%) and there was no past history of heart failure or arrhythmia. The fibrosis was a small, discrete area in the subendocardium of the mid-inferolateral wall of the LV, with normal myocardial thickness throughout and no associated regional wall motion abnormalities (figure 1).

### Mild/moderate myocardial iron loading group (T2* 10-20 ms)

In these 15 patients, the myocardial T2* was 13.1 ms (11.1, 15.4), liver T2* was 2.9 ms (2.1, 4.3) and the LV EF was 64.1 ± 6.2%. No patient had cardiac fibrosis despite 2 patients (4%) having a previous episode of heart failure. When compared with the normal group (T2* > 20), there was no significant difference in LVEF (p = 0.34).

### Severe myocardial iron loading group (T2* < 10)

In these 3 patients, the myocardial T2* was 7.7 ms, liver T2* 2.2 ms and LV ejection fraction was 54.7 ± 3.5%. All 3 patients (100%) had a previous history of heart failure, but no patient has cardiac fibrosis. When compared with the normal group (T2* > 20) the LVEF was significant reduced (p < 0.01).

## Discussion

Macroscopic cardiac fibrosis is detectable using LGE CMR and occurs commonly in non-ischemic heart disease such as dilated cardiomyopathy (DCM) [11,15], and hypertrophic cardiomyopathy (HCM) [12,17]. In DCM and HCM, the fibrosis is a driver of the development of LV dysfunction, and is directly linked to adverse cardiac outcomes such as heart failure and arrhythmias [15,17]. Historical post-mortem studies showed replacement cardiac fibrosis to be a prominent feature in thalassemia major patients [4-6], and therefore it is possible that cardiac fibrosis is important in the development of heart failure in thalassemia major. However, these studies are now up to 50 years old, which pre-dates the era of iron chelation treatment, and were biased towards patients who had died. More recent histology studies are limited and mainly in the unrelated genetic condition of hereditary hemochromatosis [7-9]. Our study in the modern chelation era of living patients, shows a low incidence of macroscopic myocardial fibrosis of 2% (1 in 45 patients), and in this one patient the fibrosis was very limited affecting only 0.4% of total left ventricular mass. This is considerably lower than the replacement fibrosis typically seen in DCM (15%) [15,11] and HCM (30%) [17], and is unlikely to have any clinical relevance as studies in myocardial infarction predict a decrement in ejection fraction of only 0.27% in association with fibrosis of 0.4% of left ventricular mass [29]. If the fibrosis was due to cardiac siderosis, myocardial iron loading should be present, but the cardiac T2* was normal at 20.1 ms; or the patient might have current or a past history of heart failure, but this was not present and the ejection fraction was normal at 57%. The 47 year old male patient had not been investigated by the referring hospital for alternative, more prevalent causes of fibrosis, such as coronary disease, but was hepatitis C antibody positive.

Another significant discrepancy related to the possibility of cardiac fibrosis causing ventricular dysfunction in thalassemia major is that the dysfunction can be reversed with successful iron chelation therapy with intravenous deferoxamine [3], or combination deferoxamine-deferiprone [30]. The left ventricular ejection fraction increases as cardiac T2* improves, however there is no obvious pathophysiological mechanism for reversible effects of replacement fibrosis on ejection fraction. When the association of improved ejection fraction and improved cardiac T2* is married with the knowledge that low cardiac T2* is a potent predictor of future cardiac events [31], it becomes difficult to ascribe a significant clinical role to another factor such as putative reversible fibrosis. The lack of replacement fibrosis in our patients who had a history of heart failure or current left ventricular impairment would add credence to this position. We posit therefore that the left ventricular impairment seen in cardiac siderosis is a direct result of myocardial iron deposition and any contribution from replacement fibrosis is limited.

There is one previous study using CMR LGE in thalassemia from Pepe et al [10], which found LGE in 28 of 115 patients (24%). There are a number of possible explanations for the discrepancy of this study with ours. The LGE techniques or the interpretation of the images or the population studied may have differed between the 2 studies. The age and mean cardiac T2* of Pepe's patients was 27.2 years and 24.5 ms, compared with 27.1 years and a median of 22.6 ms in our study, which excludes these as possible causal differences. However there was a high level of hepatitis C antibodies in Pepe's patients (71%) which may indicate an additional mechanism for LGE such as myocarditis, which might resolve the differences in LGE prevalence between the 2 studies. In our study, only 11/49 (22%) patients were hepatitis C antibody positive, which included our one patient with LGE. We also note that the quantification of mean LGE extent by Pepe et al was 3.9% which indicates that the patches of fibrosis in their study were very limited and unlikely to be clinically significant with regard to cardiac dysfunction.

## Limitations

LGE shows in-vivo macroscopic fibrosis, but interstitial fibrosis is too fine to be detected. However new techniques with T1 mapping [32], or steady state gadolinium infusions [33], to detect microscopic (non-replacement) fibrosis may prove useful. We have no tissue analysis from our study examining molecular markers of cardiac fibrogenesis. mRNA studies in ex-vivo cardiac myocytes have shown iron level dependent reductions in expression of transforming growth factor-β1 (TGF-B1), biglycan, and collagen type I, which was accompanied by a reduction in TGF-B1 bioactivity, which does not obviously support iron-driven cardiac fibrogenesis [34]. However, other studies suggest that myocytes can suppress proliferation of fibroblasts by cumulative effects on late G1 events leading to DNA synthesis, and these effects are diminished with myocyte iron accumulation [35,36]. A contemporary histology study in thalassemia major examining myocardium directly for interstitial fibrosis would be useful to add further evidence to the debate.

## Competing interests

Dr Pennell is a consultant to ApoPharma, Novartis and Siemens, and is a director and stockholder in Cardiovascular Imaging Solutions. The other authors declare that they have no competing interests.

## Authors' contributions

PK and DP designed the study, collected the data and analyzed the results. JPC and MAT designed the study, and assisted in collecting the data. All authors have read and approved the final manuscript.
